# Relationship between Oral Health Status and Oropharyngeal Dysphagia in Older People: A Systematic Review

**DOI:** 10.3390/ijerph192013618

**Published:** 2022-10-20

**Authors:** Noemie Drancourt, Nada El Osta, Nicolas Decerle, Martine Hennequin

**Affiliations:** 1 Centre de Recherche en Odontologie Clinique (CROC), Université Clermont Auvergne, F-63000 Clermont-Ferrand, France; 2 CHU of Clermont-Ferrand, Service d’Odontologie, F-63003 Clermont-Ferrand, France

**Keywords:** oropharyngeal dysphagia, oral health, xerostomia, hyposalivation, oral motricity, dental status, older people

## Abstract

The purpose of this review is to investigate the relationship between oral health status and oropharyngeal dysphagia (OD) in older people and to collect a list of oral health indicators that can enable carers and health professionals to screen for risk of dysphagia in older people during oral examinations. A systematic review was performed following the Preferred Reporting Items for Systematic Reviews and Meta-Analysis Protocol (PRISMA-P 2015) guidelines. The analysis methods and inclusion criteria were documented in a protocol published in the Prospective International Register of Systematic Reviews (PROSPERO) under the registration number CRD42020140458. A total of 19 articles published between 2002 and 2020 were retained by the search criteria for the qualitative synthesis. Eighteen studies demonstrated at least one positive association between an oral health component (dental, salivary and/or muscular) and dysphagia. This review highlights that oral health and OD are associated but was not able to determine causality. The lack of scientific evidence could be explained by the observational approach of the majority of the studies and the irrelevant choice of oral health indicators. A relationship may exist between oral health and dysphagia, but this review highlights the lack of valid and standardized oral health indicators that would be needed to assess the impact of oral health on the overall health status of individuals.

## 1. Introduction

The European Society for Swallowing Disorders and the European Union Society of Geriatric Medicine [1] have recognized oropharyngeal dysphagia (OD) as a geriatric syndrome [2] because it meets the following conditions: (a) high prevalence in older persons; (b) a combination of symptoms relating to a difficulty in forming or moving a bolus safely from the oral cavity to the esophagus (aspiration, residual, excessive throat clearing, coughing, hoarse voice, atypical ventilation periods, and repetitive swallowing); (c) common risk factors (functional dependence, cognitive dependence, age, frailty, polymedications, and multimorbidity); (d) interactions with other geriatric syndromes (stroke, dementia); (e) poor outcomes (associated with higher short- and long-term mortality, and pneumonia); (f) multidisciplinary treatment. According to a comparative table produced by Ortega et al., the prevalence of OD was 27% in community-dwelling older persons [3], 47.5% in acute-care hospitalized older patients [4,5], 51% in nursing home residents [6] and 91% in older patients hospitalized for community-acquired pneumonia [7]. However, a considerable percentage of individuals with OD are not routinely identified because they spontaneously adapt food texture to their chewing capacities and exclude hard-to-chew foods from their diet, and/or because they suffer from silent aspiration [8].

Oral health factors affecting oral food processing may comprise swallowing, resulting in OD [9,10]. Oral health refers to the health of the teeth, gums, and the entire oral-facial system that allows us to smile, speak, and chew [11]. Chewing and manipulation of food by soft tissues, as well as mixing of food with saliva, ensures the formation of a bolus of appropriate size and consistency that the tongue can transport efficiently to the oropharyngeal isthmus (narrow passageway between the pharynx and the base of the tongue) [12]. During chewing, the food is transformed into a bolus by the actions of the teeth through the exertion of lingual, facial and masticatory muscles and with the presence of saliva. The food bolus must be sufficiently well prepared to be swallowed easily and safely. It must be slippery, cohesive and plastic. Plasticity allows the bolus to deform as it passes through the digestive tract, while slipperiness facilitates transport along the mucous membranes and down the narrow alimentary canal to the stomach. Finally, cohesiveness means that the bolus must behave as a unit. This is essential to avoid oropharyngeal dysphagia, which can occur if food particles disperse and enter the airway when the bolus passes through the aerodigestive junction [13]. Three factors have a major impact on masticatory function in older individuals: (i) impairment of the motor apparatus function, (ii) quantity or/and quality of saliva, and (iii) number of natural antagonist teeth [14,15]. The impact of poor oral health on mastication [16] and malnutrition has already been demonstrated [17]. However, the scientific evidence assessing the influence of poor oral health on swallowing dysfunction is limited.

Identifying those parameters of poor oral health that increase the risk of OD is important for community-dwelling older adults, and especially for those with cognitive impairments who might have difficulty expressing their needs. OD has a major impact on the quality of life for older adults, with physiological and social consequences. The main health complications for individuals are malnutrition, dehydration and aspiration pneumonia, which lead to poor public health outcomes, such as increased length of hospital stay with readmissions, and increased morbidity and mortality [18,19]. It has been shown that food texture and oral intake restrictions in nursing home residents are not based on systematic oral examinations [20]. A wide range of oral health criteria are detailed in the literature, including psychometric, biological, physiological and anthropometric ones. However, there is a need to define the relevant oral health criteria that could be used to signal those at risk of OD.

The objective of this systematic review was (i) to investigate the association between oropharyngeal dysphagia and oral health status in older people, and (ii) to create a list of oral health indicators to enable dental and health professionals (physicians, physiotherapists, speech therapists, nurses, caregivers) to screen older people for OD risk during oral examinations.

## 2. Materials and Methods

A systematic review was carried out using the Preferred Reporting Items for Systematic Reviews and Meta-Analysis Protocol (PRISMA-P 2015) guidelines [21]. The analysis methods and inclusion criteria were laid out in a protocol published in the Prospective International Register of Systematic Reviews (PROSPERO) under the registration number CRD42020140458.

### 2.1. Search

The bibliographic search involved the PubMed^®^ and Cochrane databases. We also checked that no similar protocol already existed in PROSPERO when registering the project for this literature review. The main keywords used were “Dysphagia, Oral health, Elderly”. An advanced search was carried out using these terms for all available fields. Filters were applied to select English language texts and subjects aged 65 years or over. Records were collected using this search strategy, and any duplicates were excluded. All articles including subjects aged 65 years or older, related to good or poor oral health status, which dealt with oropharyngeal dysphagia as the clinical outcome of interest were included. During screening, studies whose titles and/or abstracts were related to subjects under the age of 65, and/or those dealing with the treatment of dysphagia or with methods of assessing dysphagia were excluded.

### 2.2. Investigators

One investigator and one supervisor carried out the pre-search using the keywords. Two investigators and one supervisor then undertook the different steps to select the articles.

### 2.3. Data Extraction

A Microsoft Excel file was created, and a pre-pilot form was tested by two investigators. All quantitative and qualitative variables were considered as items and were used as column headings while the articles themselves were listed in the rows. The articles did not necessarily contain all the items but were listed in those columns that were relevant. Finally, three groups of items were listed for data extraction:

(1) Ten items relating to the article and the study: article title; author list; journal the article was published in; year of publication; duration of the study; type of study; tool studied; place where the study was carried out; summary of the text; opening and pertinent remarks about the study.
(2) Ten items relating to the subjects of the study and general information: number of subjects; age; situation (home, hospital, etc.); general information concerning the study; specific pathologies; study of health status and comorbidities; study of nutrition; cognitive abilities; dependency; daily activities.
(3) Seven items related to dysphagia and oral health: cause of dysphagia; consequences of dysphagia; assessment methods used for identifying dysphagia (questionnaire or test); dental and periodontal criteria (study of the occlusion, number of teeth or functional units, bite force, oral hygiene, dental plaque, periodontal study, decay, presence and characteristics of prostheses); study of the oral dryness; study of the muscles (tongue, motor structures: lips, larynx); study results (impact of dental status on dysphagia, impact of the salivary or muscular state on dysphagia).

### 2.4. Assessment of the Quality of the Studies Included

Three investigators and one supervisor applied the STROBE GRADE approach (which measures confidence in the analysis of observational studies) [22] to grade the quality of the evidence in the different studies. Each investigator scored the studies that were included independently, and in the case of disagreement, discussion between the investigators was used to reach a consensual decision.

### 2.5. Strategy for Data Synthesis

After analysis, the results were synthesized into a short narrative review, targeting the relationship between oral health and OD in an elderly population. The criteria that were used for this analysis are presented in tables.

## 3. Results

### 3.1. Search Results

**Figure 1:**
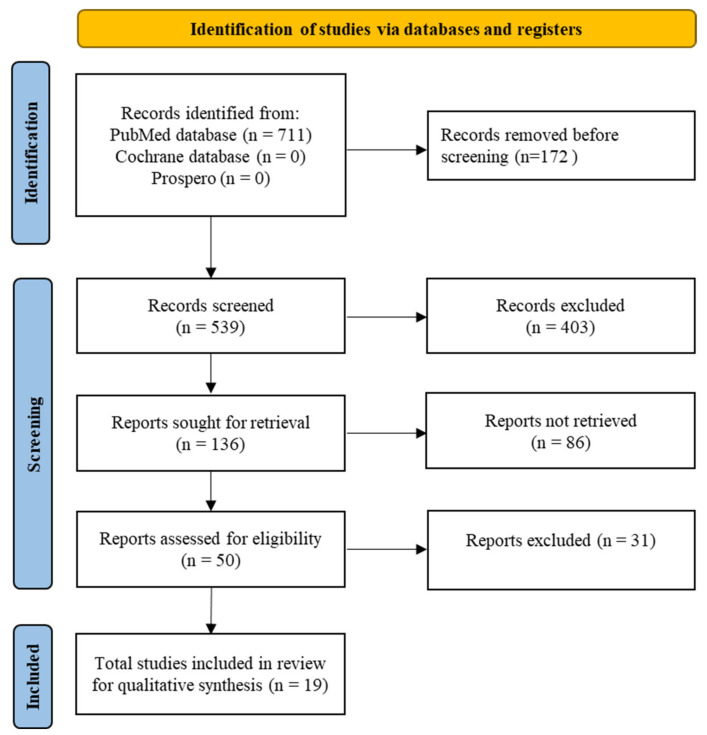
Flow chart for inclusions.

A flow chart, presenting the selection process of included records, is displayed in Figure 1, following the PRISMA 2020 flow diagram guidelines [23]. The search carried out on 28 May 2020 identified 711 records in the PubMed database, and no articles from the Cochrane Library. After applying the filters “65 years and older” and “English”, this was reduced to 539 records. No duplicates were detected, and 539 articles were evaluated by title. At this stage, 403 records were excluded, with a total of 136 records considered eligible for reading of the abstract. After analysis of the abstracts, a further 86 records were excluded based on the exclusion criteria. Of the 50 records that were eligible for full-text reading, 31 were excluded at this stage. The final search for the qualitative synthesis included 19 articles, published between 2002 and 2020.

Among the included studies, 17 were cross-sectional and two were cohort studies. The STROBE score for the included articles varied from 9.5 to 23.5. Nevertheless, all 19 studies used one or more measure to assess the presence of dysphagia, and one or more oral health indicator related to dental, muscular, or salivary aspects. Methods used to assess oropharyngeal dysphagia and oral health are shown in Table 1.

### 3.2. Study Analysis

The criteria used in the studies to establish relationships between dental status, saliva and oral motor skills and oral dysphagia are given in Table 2, Table 3 and Table 4, respectively.

#### 3.2.1. Impact of the Number of Functional Teeth, Occlusion and Chewing Function on OD

The oral health indicator most frequently assessed was the impact of the residual number of functional teeth or occlusion on OD. A significant association between teeth and OD was found in 15 studies. Cross-sectional studies were often applied, so a causal relationship could not be established.

*Number of teeth:* Six studies [26,27,34,36,37,41] reported that older adults with fewer remaining teeth were more likely to develop swallowing problems compared with those with a greater number of remaining teeth. However, this link was not found in two others studies [31,33].

*Occlusion and mandibular stability:* Eight studies investigated the impact of posterior teeth occlusion or molar occlusion on OD. The findings of five studies demonstrated that loss of *posterior teeth occlusion or the absence of mandibular stability* was significantly associated with swallowing problems and dysphagia [24,33,35,38,42]. However, this association was not found in the other three studies [26,40,41].

*Masticatory function:* One study evaluated the relationship between masticatory function and dysphagia. The findings of Rech et al. showed that a non-functional oral health status (edentulism with/without unadjusted dentures) is associated with a higher frequency of dysphagia [25].

*Use of dentures:* Two studies showed that wearing prostheses decreases the risk of dysphagia by increasing the number of functional units [28,33]. However, Brochier et al. found that prosthesis adaptation was not significantly associated with OD [33]. The study of Onodera et al. demonstrates that wearing dentures might improve the swallowing function of older individuals. They also could compensate for denture absence by expanding the range of oropharyngeal movements during pharyngeal swallowing to ensure the smooth passage of the bolus throughout the pharynx [30].

#### 3.2.2. Impact of Hyposalivation, Xerostomia and Oral Candidiasis on Oral Dysphagia

Six studies found a significant association between dry mouth sensation or low salivary flow and dysphagia. These findings revealed that older people who complained of dry mouth symptoms [29,33,36,39,41] or those who experienced low salivary flow [42] were more likely to experience difficulty in swallowing function compared to non-dry mouth individuals.

Xerostomia inventory [33], the visual analog scale [39], the presence or absence of subjective oral dryness [26,29,36,41], clinical diagnosis of mouth dryness [40], and a salivary test using a sterile compress under the tongue [42] were used for the diagnosis of xerostomia or salivary insufficiency.

However, two others studies found no significant relationship between swallowing problems and the perception of a dry mouth [26,40].

Only one study evaluated the relationship between oral candidiasis and dysphagia in older individuals and found a significant positive association [42].

#### 3.2.3. Impact of Orofacial Motor Skills and Tongue Motricity on Oral Dysphagia

Six studies investigated the relationship between orofacial motor skills and dysphagia. The first study found that individuals who had modifications to four or more oral sensorimotor functions presented a higher prevalence of dysphagia in community-dwelling older people and for long-term care residents [25]. Two studies found that the loss of tongue strength [25] and poor tongue mobility [40] were significantly associated with decreased swallowing function. The findings of the third study revealed that individuals with lower swallowing pressure were more likely to be classified as having dysphagia than those with a higher pressure (>26 kPa). However, maximum isometric tongue-pressure (measured by a series of three lingual presses on the anteriorly placed bulb) was not related to dysphagia [32]. Okamoto et al., reported in their first study that the maximum bite force was lower in subjects with swallowing problems compared to those with no swallowing problems [41] but the results were not reported in their second study because maximum biting force was not included in the multivariate analysis (Okamoto et al., 2015 [26]). Nishida et al., 2020 found that participants with impaired chewing ability were more prone to presenting swallowing problems than others (Nishida et al., 2020 [29]).

### 3.3. Narrative Review

In this review, of the 19 articles included, 18 studies showed at least one positive association between an oral health component (dental, salivary and/or muscular) and dysphagia. However, only two follow-up surveys [26,41] and one observational study [40] evaluated the relationship between OD and oral status using each of the three dental, xerostomia and oral motricity parameters. The multiplicity of the oral health indicators selected for the 19 studies could affect the validity of the results. In addition, of the studies conducted to assess the association between OD and oral health outcomes, only 11 studies were designed to consider the strength of this association after controlling for other variables [24,25,26,27,28,29,33,36,38,39,41]. As a result, although this review highlights that oral health and OD are associated, it is not possible to determine the causality.

The lack of scientific evidence could be explained by the observational approach of the majority of studies and the irrelevant choice of oral health indicators. Numerous oral health indicators were used, and in addition the methods used to collect the criteria vary considerably. Objective criteria were reported in all 16 dental assessment studies [24,25,26,27,28,30,31,33,34,35,36,37,38,40,41,42] and in five of the six studies assessing oral motor skills [25,26,32,40,41]. However, only two of the eight studies examining the association between OD and hyposalivation used objective criteria [40,42]. In addition, the method used for measuring objective and subjective criteria was not described in detail in some studies [24,26,27,28,31,33,34,36,37,38,41,42], or was inconsistent [25,26,29,33,40]. Therefore, given the current stage of the literature, it is not possible to carry out a meta-analysis to estimate the overall link between OD and oral health. For this, additional studies designed to assess relevant oral health indicators specifically related to swallowing would need to be performed. Prospective studies using standardized and validated indicators are also needed to establish evidence-based relationships between OD and oral health.

## 4. Discussion

This review shows that oral status and oropharyngeal dysphagia are related in older people. It highlights the importance of choosing pertinent oral health indicators for further studies. In particular, dental status has an impact on dysphagia and in turn on food choices, malnutrition and sarcopenia. In older people, poor dental health could contribute to the development of sarcopenic dysphagia due to the loss of mass and strength of the muscles involved in swallowing [43].

The main problem in screening for the relationship between oral health and OD was related to the selection of the oral health variables, including the dental status indicators. The total number of residual teeth in the mouth, the number of decayed or missing teeth, periodontal status, and the level of oral hygiene are not accurate measures for assessing an individual’s chewing ability and swallowing problems, although they may indirectly affect them. In order to examine the association between oral health and OD, it is crucial that the selected criteria effectively measure the contact between posterior antagonist teeth. During mastication, it is these inter-arch contacts that progressively fragment food, allowing it to be transformed into a swallowable bolus once mixed with saliva. Inter-arch dental contacts are vital for providing isometric co-contractions of masticatory muscles acting on the jaw during swallowing. In turn, co-contractions of the suprahyoid and infrahyoid muscles stabilize the hyoid bone during laryngeal traction. Moreover, with aging, there is a change in the swallowing mechanism, with a reduction in mobility, strength, and sensitivity, in addition to changes in the oral mucosa [25]. For these reasons, oral motricity could be affected by dental status.

Depending on the study, teeth that participate in mastication and swallowing are called “functional teeth” [25,28], “functional dental units” [38], “posterior molar occlusion” [40], “number of occluding pairs” [33], “posterior teeth occlusion” [38], “posterior occluding pairs” [42] or “occlusal support” [26,33,35,38,40,41,42]. Different methods were used to identify the number of functional pairs of teeth. In some studies, it was shown on an odontogram after a clinical examination and the number of functional units was calculated based on the attribution of different coefficients to molars and premolars [26,35,41,44,45]. However, this method is theoretical and does not consider malposition of the teeth, dysmorphology, jaw dyspraxia, or denture use. In other studies, posterior occlusion was used as a criterion for masticatory ability but was evaluated differently; posterior occlusion was determined according to the presence of pairs between molars and/or premolars, with an occlusal pair defined as any type of contact between antagonist teeth, both natural and artificial [33,38,42,46]. In a different study, posterior molar occlusion was examined from the first premolar to the second premolar with natural teeth and dentures, and groups were defined as molar occlusion groups and no occlusion groups [40].

A simple method to check for posterior functional pairs of teeth is described in mastication and/or nutrition studies [47,48,49,50], but was not measured in these selected articles. In this method the masticatory function of subjects with natural teeth/or fixed prostheses, and of those who had worn their dentures for the last two meals, is assessed by asking the participants to chew for 1–2 cycles on 200 µm thick articulating paper to register the number of posterior dental functional units (PFUs). For participants who have dentures but did not use them for the last two meals, the test was performed without dentures. The number of PFUs is given by the number of posterior teeth (either natural or prosthetic) on the mandibular arch that had at least one colored mark. By convention, the number of posterior teeth in the maxilla with colored marks is not counted. When tThe number of PFUs lies between 0 and 10, and masticatory efficiency is considered to be affected when it is less than, or equal to four.

In our systematic review, only one study found a negative association between dysphagia and oral health [31]. In this study, patients with signs of dysphagia had a significantly higher mean number of teeth (7.26 ± 7.64) than those without dysphagia (2.00 ± 3.1), but the authors did not give a clear reason for this association.

Some studies suggested that gender could be a cofactor in the relationship between the number of functional teeth and oral dysphagia. According to Fukai et al., the critical functional tooth number (CTN) required for prevention of subjective dysphagia caused by oral impairment decreased with age in both men and women; however, women showed a slightly higher CTN than men. At 80 years old, women are at major risk of dysphagia if they have less than four teeth, and men are at major risk of dysphagia if they have less than 10.1 teeth [28]. The findings of Furuta et al. also showed that women have fewer teeth than men, yet they tend to have better swallowing function [34].

Conversely, Wang et al. found that women were more likely to have problems with chewing and swallowing compared to men [27]. The findings of Nishida et al. exposed that dysphagia was independently associated in women. In their discussion, Nishida et al. stated that women might be more sensitive to poor oral health and swallowing difficulties compared to men, and that differences in the care-seeking behavior in the two genders might have affected the results. They then set out the need for an objective screening test to confirm gender differences [29].

A potential limitation of this systematic review is the search criteria. The choice of three databases and keywords could not guarantee a full coverage of the search. The inclusion of articles in the English language only, the search equation (keywords and wording) and the filters applied for subjects aged 65 years or more may have led to the non-selection of certain references, the exclusion of others and the search was not exhaustive, particularly in terms of those studies in which “age” was not referenced. The definition of an “older person” differs in the literature, although the most commonly used age to define an elderly person in medical research is 65 years [51]. Therefore, the cut-off age of 65 years was chosen as the reference standard in our systematic review to define an “elderly” population. Additionally, since this review was limited to studies published in English, relevant scientific studies published in other languages might be missing. Searches were not carried out in the gray literature because of the multiplicity of sources and the difficulty of exhaustiveness, which can lead to publication bias. Finally, the bottom-up approach was not applied to the included articles.

This systematic review disclosed considerable clinical heterogeneity, i.e., differences between studies mainly in the methods used for assessing dysphagia, which makes it difficult to compare articles. In addition, three articles had a STROBE score of less than twelve [24,30,37], so the test for evidence of association was based on quite a low level of evidence. Sample sizes also vary considerably between studies, and the necessary number of subjects was rarely calculated. Lastly, the lack of control checks for confounding variables in some studies could affect the validity of the results.

## 5. Conclusions

Despite the small number of studies evaluating the relationship between oral health status and dysphagia in people over 65 years of age, this systematic review suggests that a relationship may exist between these two parameters. However, the review highlights the lack of valid and standardized oral health indicators that could be used to assess the impact of oral health on the overall health status of individuals. This lack of scientific evidence may contribute to misinterpretations and misunderstandings that could compromise the quality of life of older people who complain of swallowing disorders or pathologies associated with dysphagia. The number of functional pairs of teeth and/or the occlusion (natural or prosthetic), the individual’s perception of mouth dryness, and the tongue motor skills could be indicators for detecting oropharyngeal dysphagia in older people. However, these indicators should be collected using objective and valid criteria based on clinical arguments.

## Figures and Tables

**Table 1:** Title, year of publication, STROBE score, methods of evaluation, and oral health assessment criteria of included articles (NE: Not evaluated; O: Objective assessment; S: Subjective assessment).

References	Titles	Score Strobe	Dysphagia Assessment	Oral Health Assessment		
				Oral Motricity	Xerostomia	Dental Status
Tamura F. et al., 2002 [24]	Analysis of feeding function and jaw stability in bedridden elderly	9.5	O	NE	NE	O
Rech R et al., 2018 [25]	Association between oropharyngeal dysphagia, oral functionality, and oral sensorimotor alteration	18.5	O	O	NE	O
Okamoto N et al., 2015 [26]	Association of tooth loss with the development of swallowing problems in community-dwelling independent elderly population: The Fujiwarakyo study	21	O + S	O	S	O
Wang TF et al., 2012 [27]	Associations between chewing and swallowing problems and physical and psychosocial health status of long-term care residents in Taiwan	22.5	S	NE	NE	O
Fukai K et al., 2011 [28]	Critical tooth number without subjective dysphagia	12	S	NE	NE	O
Nishida T et al., 2020 [29]	Dysphagia is associated with oral, physical, cognitive and psychological frailty in Japanese community-dwelling elderly persons	20	S	S	S	NE
Onodera S et al., 2016 [30]	Effects of wearing and removing dentures on oropharyngeal motility during swallowing	10.5	O	NE	NE	O
Bomfim et al., 2013 [31]	Factors associated with suggestive signs of oropharyngeal dysphagia in institutionalized elderly women	15.5	O	NE	NE	O
Namasivayam-MacDonald AM et al., 2017 [32]	How swallow pressures and dysphagia affect malnutrition and mealtime outcomes in long-term care	22	O	O	NE	NE
Brochier CW et al., 2018 [33]	Influence of dental factors on oropharyngeal dysphagia among recipients of long-term care	19	O	NE	S	O
Furuta M et al., 2013 [34]	Interrelationship of oral health status, swallowing function, nutritional status, and cognitive ability with activities of daily living in Japanese elderly people receiving home care services due to physical disabilities	23.5	O	NE	NE	O
Wakabayashi H et al., 2018 [35]	Occlusal support, dysphagia, malnutrition, and activities of daily living in aged individuals needing long-term care: a path analysis	21.5	O	NE	NE	O
Inui A et al., 2017 [36]	Oral conditions and dysphagia in Japanese, community-dwelling middle- and older-aged adults, independent in daily living	17	O + S	NE	S	O
Ortega O et al., 2014 [37]	Oral health in older patients with oropharyngeal dysphagia	9.5	O + S	NE	NE	O
Okabe Y et al., 2017 [38]	Posterior teeth occlusion and dysphagia risk in older nursing home residents: a cross-sectional observational study	23	O	NE	NE	O
Ohara Y et al., 2011 [39]	Ratio and associated factors of dry mouth among community-dwelling elderly Japanese women	17.5	O + S	NE	S	NE
Murakami K et al., 2015 [40]	Relationship between swallowing function and the skeletal muscle mass of older adults requiring long-term care	14.0	O	O	O	O
Okamoto N et al., 2012 [41]	Relationship between swallowing problems and tooth loss in community-dwelling independent elderly adults: The Fujiwara-Kyo study	21.5	O + S	O	S	O
Poisson P et al., 2016 [42]	Relationships between oral health, dysphagia and undernutrition in hospitalized elderly patients	21	O	NE	O	O + S

**Table 2:** Criteria used to establish a link between dental status and dysphagia.

Articles	Assessment of Dysphagia	Assessment of Dental Status	Relationship between Dysphagia and Dental Status
Tamura F. et al. (2002) [24]	**Subjective evaluation**: NE	- Dental examinations by four dentists. Criterion of the **jaw stability**: ability to achieve posterior occlusal contact with natural dentition or prosthesis under an appropriate occlusal guidance. Patients were categorized as having mandibular stability or not having mandibular stability.	Swallowing frequency value for patients with mandibular stability was greater compared to those with unstable mandible. Duration for onset of first swallow was greater in patients with unstable mandible compared to those with unstable mandible. Evaluation of feeding function during a meal was not significantly different between subjects having or not having mandibular stability.
	**Objective evaluation**: - Repetitive saliva swallowing test (RSST) (unless cognitive problems or tube-feeding) (speech-pathologist): counting the frequency of swallowing over a 30 s period. - Water swallowing test: 15 mL water in a cup to swallow (physician). Swallowing behavior observed and described. - Observatory evaluation of feeding function during a meal: signs of choking and coughing, lip function, and duration from food intake into the oral cavity until swallowing.		
Rech et al. (2018) [25] xx]	**Subjective evaluation**: NE	Oral status by one dentist according to the criteria of the World Health Organization: **Categorization of oral health status**: - Functional: all natural teeth or partial tooth loss rehabilitated with an adjusted partial dental prosthesis. - Partially functional: partial tooth loss without dental prosthesis rehabilitation or edentulous with adjusted complete dentures. Non-functional: edentulous, edentulous with unadjusted complete dentures or partial tooth loss with unadjusted dental prosthesis.	Individuals with a non-functional oral health status presented a higher prevalence of dysphagia.
	**Objective evaluation** by a specialist speech-language therapist - Indirect swallowing test and direct deglutition test assessing the three food consistencies (pasty, liquid, and solid). - Anatomy and physiology (masticatory efficiency, time of bolus formation, efficient swallowing) - Clinical signs and symptoms of laryngotracheal penetration or aspiration (coughing, choking, food stuck or stopped in the throat, vocal change, and food discomfort) - Cervical auscultation		
Okamoto N. et al. (2015) [26]	**Subjective assessment**: Do you drop food from your mouth during a meal? (yes/no) Do you feel that food remains in your mouth? (yes/no) Do you choke during a meal? (yes/no) Do you cough during and after a meal? (yes/no)	- Calibration of dental examinations by two dentists: - **Number of remaining teeth** defined as healthy, carious, or treated (including crowned, inlay, and abutment teeth for bridge), inclusive of completely erupted third molars. Root tips and very loose teeth indicated for extraction were not included as remaining teeth. - **Occlusal support**, including artificial teeth in bridges and dentures, according to the Eichner classification based on the presence or absence of occlusal contact in the posterior area. The region is divided into four support zones, two in the premolar and two in the molar regions: A (four support zones posteriorly), B (one to three support zones posteriorly or the presence of occlusal contacts anteriorly), and C (no occlusal contact on the remaining teeth).	The incidence of swallowing problems was significantly greater with fewer remaining teeth. The occlusal support was not significantly related to dysphagia
	**Objective assessment** by two trained dentists and four dental hygienists 30 mL water swallowing test, followed by a discussion of the observations to achieve a consensus.		
Wang T.-F. et al. (2012) [27]	**Subjective assessment** Not clearly addressed, it concerns “problems chewing and swallowing” (PCS) that is just checked by nurses on the questionnaire, but it is not clear if it is the patient’s or nurse’s opinion.	- Had **dentures or removable bridge** - **Some natural teeth remaining:** ND - **Broken, loose, or carious teeth:** ND	- Having dentures was not associated with swallowing problems - Residents who retained some natural teeth were less likely to have swallowing problems. - Residents with broken, loose, or carious teeth were more likely to have swallowing problems
	**Objective assessment**: NE		
Fukai K. et al. (2011) [28]	**Subjective assessment** Subjective dysphagia (yes/no) defined as suffering any kind of subjective impairment to eating function such as biting difficulty, swallowing difficulty caused by tooth loss, no fitted dentures or other oral impairments.	Dental health examination performed by dentists: - **Number of functional teeth with and without dentures**: ND	The minimum number of functional teeth needed to avoid subjective dysphagia might not be as high as in young people.
	**Objective assessment**: NE		
Onodera S. et al. (2016) [30]	**Subjective assessment**: NE	**Wearing full dentures or not**	Oropharyngeal movements during pharyngeal swallowing vary with and without dentures. Spatial change of oropharyngeal movement to avoid temporal changes in pharyngeal swallowing when dentures were absent in edentulous older individuals.
	**Objective assessment**: Videofluorography with solid test food (minced agar jelly 40% barium sulphate, particle diameter of 40–56 mm). Texture of test food adjusted to be masticated and swallowed with dentures and without dentures → quantitatively evaluated range, distance and duration of oropharyngeal movements during pharyngeal swallowing.		
Bomfim et al., 2013 [31]	**Subjective assessment**: NE	- **Number of teeth**: ND - **State of dental conservation** (adequate/inadequate): ND - **Use of dental implant**: ND - **Use of upper dental implant**: ND	Mean number of teeth greater in case of suggestive OD State of dental conservation and use of upper dental implant were not associated with suggestive OD
	**Objective assessment** The signs suggestive of oropharyngeal dysphagia based on the Dysphagia Risk Evaluation Protocol: front oral escape, food residue after deglutition, voice change after deglutition, vocal change after deglutition, increased oral phase, spitting food or saliva, biting the utensil, antagonistic tongue movement at the entrance of food, coughing during meals, choking, fatigue/ respiratory changes, altered cervical auscultation. The presence or absence of each of the signs indicating oropharyngeal dysphagia.		
Brochier CW. et al. (2018) [33]	**Subjective assessment**: NE	Clinical dental examination by one dentist: - **Number of occluding pairs**: none, 1–7, 8–14, prosthetic pairs. ND - **Number of teeth**. ND - **Assessment of dental prosthesis in accordance with the number and type of prosthesis**: partial removable prosthesis, complete denture, single or multiple fixed prosthesis **Retention**: using the traction of the index finger on the palatal of the anterior teeth **Stability**: pressuring a point of the hemiarch and the existence, or not, of an elevation of the adjacent hemiarch **Capacity to injure issues**: swollen or reddish injuries The prostheses were classified as adapted, slightly maladapted, partially maladapted and totally maladapted.	- Older persons with no occlusal pairs had the highest prevalence of oropharyngeal dysphagia, when compared to older persons with 8 to 14 mixed pairs. - Number of prostheses, prosthesis adaptation, and number of teeth were not associated with OD.
	**Objectiveassessment** Clinical assessment of deglutition with two stages: - Indirect swallowing test (saliva swallowing, forced coughing, anatomical conditions) - Direct swallowing test evaluating food consistencies (liquid, semi-solid, solid). Clinical signs/symptoms of laryngotracheal penetration or aspiration: masticatory efficiency, time of bolus formation, efficient deglutition, coughing, asphyxia, food stuck or stopped in the throat, voice change and food discomfort. - Cervical auscultation for all consistencies to disregard dysphagia, all of the items assessed had to be considered normal.		
Furuta M. et al. (2013) [34]	**Subjective assessment**: NE	Oral health assessment by qualified dental hygienists. - **Number of teeth**: 0–9; 10–19; ≥20 ND - **Denture wearing**: not wearing; wearing ND	Having many teeth and wearing dentures promoted normal swallowing function. Chewing difficulties resulting from having fewer teeth and no dentures can lead to dysphagia.
	**Objective assessment** by qualified dental hygienists Cervical auscultation: listening with a stethoscope to the sounds of swallowing 3 mL of water during the pharyngeal phase: stridor, coughing, or throat clearing considered as impaired swallowing function		
Wakabayashi H. et al. (2018) [35]	**Subjective assessment**: NE	**Occlusal support with modified Eichner index**: Occlusal contacts in premolar and molar regions on each side with natural teeth or dentures. There are four posterior support zones: the left molar, left premolar, right premolar, and right molar regions. Occlusal contacts are categorized into three classes. Class A (occlusal contacts in all four posterior support zones), class B (one to three posterior support zones or support in the anterior teeth only), and class C (no occlusal contacts). Participants classified were into two groups based on occlusal support function: functional (class A) and non-functional occlusal support groups (Eichner index classes B and C).	Occlusal support was associated directly with dysphagia. Occlusal support affects both mastication and swallowing functions, as chewing movements are necessary to eat solid food. Therefore, encouraging denture wearing to achieve occlusal support when teeth are missing is important to improve swallowing function.
	**Objective assessment** - point ordinal Dysphagia Severity Scale (DSS) score 1, indicates saliva aspiration; 2, food aspiration; 3, water aspiration; 4, occasional aspiration; 5, oral problems; 6, minimal problems; 7, within normal limits. Scores 5 and 6 imply dysphagia without aspiration and scores 1–4 dysphagia with aspiration. One research coworker determined the DSS score by observing eating abilities, water swallowing tests, food swallowing tests, cervical auscultation, and pulse oximetry.		
Inui A. et al. (2017) [36]	**Subjective assessment** “Do you sometimes choke on drinks/food such as tea and soup?”	Dental examinations conducted by trained and experienced dentists: - **Number of healthy, carious or treated teeth** ND	The risk of dysphagia was associated with a lesser number of teeth in males. The association was not significant in women.
	**Objective assessment** Repetitive saliva swallowing test (RSST): perform saliva swallows (dry) as many times as possible in 30 s. If unable to perform three consecutive swallows → dysphagia associated with aspiration.		
Ortega O. et al. (2014) [37]	**Subjective assessment** Eating Assessment Tool (EAT-10): 10 items evaluate the severity of dysphagia symptoms. Each item is composed of a five-point Likert scale (0: no problem to 4: severe problem) with total score ranges from 0 to 40, with higher scores indicating severe dysphagia symptoms; A score of 3 or more is considered at risk for dysphagia.	Dental examination by two periodontists: - **Number of teeth** ND - **Caries** were assessed at each dental surface (four surfaces for incisors and canines; five for premolars and molars) to measure the percentage of teeth with caries and surfaces affected. - **Edentulism** and the need for dentures to eat ND - **Periodontal diseases** (debris and calculus indices)	OD patients had more caries, more edentulism and periodontal diseases.
	**Objective assessment** Videofluoroscopy (VFS) for patients with swallowing complaints. VFS signs of impaired safety were classified according to Penetration–Aspiration Scale; swallowing of series of 5 mL, 10 mL and 20 mL of nectar, liquid and viscosity pudding.		
Okabe Y. et al. (2017) [38]	**Subjective assessment**: NE	By one trained dentist - **Number of remaining natural teeth**: edentulous, 1–9 teeth, >10 teeth. Not significant. ND - **Posterior teeth occlusion**: total number of functional tooth units (total-FTUs) = number of pairs of opposing posterior natural teeth and artificial teeth in bridges, dentures, or dental implants, excluding carious teeth with extensive coronal destruction. Two opposing premolars were defined as one FTU Two opposing molars were defined as 2 FTUs. Complete dentition was defined as 12 FTUs (except for the third molars).	Subjects with dysphagia risk had a significantly lower number of total FTUs than those without dysphagia risk. Number of remaining natural teeth was not associated with dysphagia.
	**Objective assessment** by trained dental hygienist Modified water swallowing test (MWST): 3 mL of cold water to swallow injected onto the floor of the mouth using a 5 mL syringe. Score 1 to 5 1 inability to swallow with choking and/or breathing changes, 2 swallowing occurred, but with breathing changes 3 swallowing occurred with no breathing changes, but with choking and/or wet hoarseness 4 swallowed successfully with no choking or wet hoarseness 5 additional deglutition dry swallowing) occurred more than twice within 30 s). Score < 3 indicated a risk of dysphagia.		
Okamoto N. et al. (2012) [41]	**Subjective assessment** Do you drop food from your mouth during a meal? Do you have the feeling that food remains in your mouth? Do you choke during a meal? Do you cough during and after a meal?	Dental examinations by one calibrated dentist - **Number of remaining teeth** categorized into 0–13, 14–24, 25–32 teeth. ND The remaining teeth were defined as healthy, carious, or treated (including crowned, inlay, and abutment teeth for bridge), inclusive of completely erupted third molars. Root tips and very loose teeth that needed to be extracted were not included as remaining teeth. - **Intermaxillary support**, which includes artificial teeth in bridges and dentures, was evaluated according to the **Eichner classification**: the premolars and molars are counted as one region, with a total of four supporting zones. Individuals classified as rank A had four occlusal contacts in the posterior region. Rank B or C refers to zero to three occlusal contacts in the posterior region.	The prevalence of swallowing problems was significantly greater with fewer teeth but was not associated with the Eichner classification.
	**Objective assessment** 30 mL room temperature water swallow test without interruption from a cup in a seated position. Observation of the time needed to drink water and the presence or absence of choking. Normal: drink water in ≤5 s without interruption or choking. Abnormal: drink water with interruptions or with choking, or longer than 5 s. By two trained dentists and the examiners discussed their observations to arrive at a consensus.		
Poisson P. et al. (2016) [42]	**Subjective assessment**: NE	Dental examination by one dentist **DMFT index** (decayed, missing, filled teeth), **posterior occluding pairs** (POPs) ND and **dental treatment need** ND **Oral self-care autonomy** (alone, needs help).	Oral self-care dependency and having fewer than 7 POPs were related to dysphagia.
	**Objective assessment** Water test: swallow four times with increasing volumes of liquid. The first liquid is water and then water plus increasing thickening after the first sign of dysphagia (orange juice consistency, nectar juice and jelly). Test considered abnormal if the patient coughs during the test or during the first minute following the test, or if voice changes. Done twice at one-week intervals.		
Murakami K et al. (2015) [40]	**Subjective assessment**: NE	**Posterior molar occlusion** is the occlusal support region from first premolar to second molar. Groups (A): molar occlusion with remaining teeth; (B): participants who required dentures to maintain occlusion; (C): without dentures/without molar occlusion. Groups A and B were defined as the molar occlusion group, and group C was defined as the no occlusion group.	No association between swallowing function and the presence or absence of molar occlusion in multivariate analysis was found.
	**Objective assessment** Modified water swallowing test (MWST): 3 mL of cold water to swallow injected onto the floor of the mouth. Score 1 to 5 (1 is inability to swallow with choking and/or breathing changes, and 5 is additional deglutition occurred more than twice within 30 s. Score ≤ 3 indicated a risk of dysphagia. - Cervical auscultation		

NE: Not Evaluated; ND: Not described.

**Table 3:** Criteria used to establish a link between xerostomia/hyposalivation and dysphagia.

Articles	Assessment of Dysphagia	Assessment of Xerostomia/Hyposalivation	Relationship between Dysphagia and Xerostomia/Hyposalivation
Nishida T. et al. (2020) [29]	**Subjective evaluation**: Have you choked on tea or soup recently? (yes/no)	**Subjective question** “Are you concerned with being thirsty?” (yes/no)	Participants with dry mouth perception were more prone to present swallowing problems compared to others.
	**Objective evaluation**: NE		
Brochier CW. et al. (2018) [33]	**Subjective evaluation**: NE	**Xerostomia Inventory (XI)** with 11-item rating scale representing the severity of chronic Xerostomia: “I ingest liquids to help with swallowing”; “I have a feeling of dry mouth when I eat”; “I wake up during the night to drink water”; “I feel my mouth is dry”; “I struggle to eat dry foods”; “I eat sugary food to diminish the feeling of dry mouth”; “I struggle to eat certain foods”; “I feel that my facial skin is dry”; “I feel that my eyes are dry”; “I feel that my lips are dry”; “I feel that the inside part of my nose is dry” on a frequency scale of events (never, rarely, occasionally, often, very frequently).	Older persons, who presented a highest score in the Xerostomia analysis, presented a high prevalence of oropharyngeal dysphagia.
	**Objective evaluation**: -Assessment of oral sensory-motor system (lips, tongue, soft palate, mandible and larynx). - Indirect swallowing test (saliva swallowing, forced coughing, anatomical conditions) - Direct swallowing test evaluating food consistencies (liquid, semi-solid, solid). - Clinical signs/symptoms of laryngotracheal penetration or aspiration: time of bolus formation, efficient deglutition, coughing, asphyxia, food stuck or stopped in the throat, voice changes and food discomfort. - Cervical auscultation Presence of dysphagia: at least one of the alterations listed above.		
Inui A. et al. (2017) [36]	**Subjective evaluation** “Do you sometimes choke on drinks/food such as tea and soup?”	**Subjective oral dryness** (yes/no)	Individuals with oral dryness at risk of OD compared to others.
	**Objective assessment**: Repetitive saliva swallowing test (RSST)		
Ohara Y. et al. (2011) [39]	**Subjective evaluation** “Do you choke when drinking tea or soup?”	“**Does your mouth feel dry**?”; visual analog scale (VAS) ranging from 0 (not dry) to 100 (extremely dry) Subjects were categorized into a dry mouth group who complained of dry mouth and a non-dry mouth group who did not.	Individuals with dry mouth were more likely to have difficulty in swallowing compared with non-dry mouth individuals.
	**Objective assessment**: Repetitive saliva swallowing test (RSST)		
Okamoto N. et al. (2012) [41]	**Subjective evaluation** Do you drop food from your mouth during a meal? Do you have the feeling that food remains in your mouth? Do you choke during a meal? Do you cough during and after a meal?	**Subjective oral dryness** (yes/no)	The prevalence of swallowing problems was significantly greater in those without oral dryness.
	**Objective assessment** by two trained dentists and examiners discussed their observations to achieve consensus. 30 mL room temperature water swallow test without interruption in a seated position. Observation of the time needed to drink water and the presence or absence of choking. Normal: drink water in ≤5 s without interruption or choking Abnormal: drink water with interruptions or with choking, or longer than 5 s		
Poisson P. et al. (2016) [42]	**Subjective evaluation**: NE	**Salivary insufficiency**: placing a sterile compress weight 0.30 g, under the tongue for 5 min. Salivary insufficiency if weight of compress < 0.35 g (salivary flow < 0.1 g/min).	Oral candidiasis and low salivary flow were related to dysphagia.
	**Objective assessment** Water test: swallow four times with increasing volumes of liquid. The first liquid is water and then water plus increasing thickening after the first signs of dysphagia (orange juice consistency, nectar juice and jelly). Test considered abnormal if the patient coughs during the test or during the first minute following the test, or if voice changes		
Murakami K et al. (2015) [40]	**Subjective evaluation**: NE	**Mouth dryness** was evaluated according to the **clinical diagnosis** classification scale of the condition of the tongue mucosa: non-dry mouth (0), saliva exhibits viscosity (1), saliva exhibits tiny bubbles on the tongue (2), and dry tongue without viscosity and little or no saliva present (3). Dry mouth categorized as grades 1–3, whereas the absence of dry mouth was defined as grade 0.	Absence of significant association.
	**Objective assessment** - Modified water swallowing test (MWST): 3 mL of cold water to swallow injected onto the floor of the mouth. Score 1 to 5 (1 is inability to swallow with choking and/or breathing changes, and 5 is additional deglutition occurred more than twice within 30 s. Score ≤ 3 indicated a risk of dysphagia. - Cervical auscultation		
Okamoto N. et al. (2015) [26]	**Subjective assessment** Do you drop food from your mouth during a meal? Do you feel that food remains in your mouth? Do you choke during a meal? Do you cough during and after a meal?	**Subjective oral dryness** (yes/no)	Oral dryness was not related to swallowing problems.
	**Objective assessment** by two trained dentists and four dental hygienists with a discussion to arrive at a consensus. 30 mL water swallowing test		
	**Objective evaluation**: NE		

NE: Not evaluated.

**Table 4:** Criteria used to establish the link between oral motor skills and dysphagia.

Articles	Assessment of Dysphagia	Assessment of Oral Motor Skills	Relationship between Dysphagia and Oral Motor Skills
Rech R. et al. (2018) [25]	**Subjective evaluation**: NE	Sensorimotor alteration was evaluated by **clinical examination** of the: - **lips** (sealing, protrusion, retraction, rapid protrusion and retraction, diadochokinesis, strength, sensitivity), - **tongue mobility** (protrusion, retraction, left lateralization, right lateralization, rapid lateralization, tongue on the left cheek, tongue on the right cheek, tip lift, tip depression), - **tongue strength** (tip of the tongue by pushing the spatula, left side of tongue pushing spatula, right side of the tongue pushing the spatula, tongue on the left cheek with counter resistance of the finger, tongue on the right cheek with counter resistance of the finger, lifting the back of the tongue with a spatula) - **tongue sensitivity** (left anterior third, right anterior third, left middle anterior third, right middle anterior third, left posterior third, right posterior third), - **soft palate** (middle line deviation, elevation, diadochokinesis, left sensitivity, right sensitivity), - **jaw** (mouth opening, diadochokinesis, lateralization), - l**arynx** (vocal quality, voluntary cough, vocal height, vocal intensity, maximum phonation time, laryngeal movement during phonation, laryngeal movement during swallowing, count from 1 to 10). - **sensorimotor alteration** was classified according to 0–1, 2–3, or 4 or more components altered. The protocol tested and validated by a pilot study.	Individuals who had four or more oral sensorimotor alterations presented a higher prevalence of dysphagia.
	**Objective evaluation** by a specialist speech-language therapist - Indirect swallowing test and direct deglutition test assessing the three food consistencies (pasty, liquid, and solid). - Anatomy and physiology (masticatory efficiency, time of bolus formation, efficient swallowing) - Clinical signs and symptoms of laryngotracheal penetration or aspiration (coughing, choking, food stuck or stopped in the throat, vocal change, and food discomfort) - Cervical auscultation		
Namasivayam-MacDonald AM et al. (2017) [32]	**Subjective evaluation**: NE	**Measures of tongue strength** using the Iowa Oral Performance Instrument (IOPI). The IOPI is a handheld pressure bulb system that consists of a small air-filled bulb, squeezed between the tongue and the hard palate. A strain gauge sensor inside the device measures the amount of air displaced from the bulb in kilopascals. - **Maximum anterior isometric tongue pressures** (MIPs) recorded across a series of three bulb squeezes, with the bulb held in an anterior position, just behind the teeth. - **Saliva swallows** were recorded across a series of three cued tasks, with the bulb held in the same anterior position. **Tongue-pressure tasks** were cued with a 10 s rest between task repetitions. In total, 2 min were required to collect the tongue-pressure measurements.	Maximum anterior isometric tongue pressure: was not different between participants with and without suspected dysphagia. Maximal swallowing pressures were lower in residents classified as having suspected dysphagia compared to those without.
	**Objective evaluation**: Dysphagia status is a composite variable. - Receiving thickened liquids. - Swallow screen using the Screening Tool for Acute Neuro Dysphagia (STAND). Consumption of three teaspoons of applesauce and 90 mL of water with signs of coughing, wet voice quality, throat clearing. - Observation of coughing or choking across any of the three meals.		
Murakami K et al. (2015) [40]	**Subjective evaluation**: NE	**Tongue mobility**: move the tongue from side-to-side (i.e., left to right). Participants who could not obey instructions, were examined by an investigator who stuck out their own tongue and asked the participant to imitate this action. If a participant’s proglossis could pass beyond the dental arch and they could move their tongue from side-to-side, their tongue motility was defined as good; all other participants were defined as having poor tongue motility.	Poor tongue motility was significantly correlated with decreased swallowing function.
	**Objective assessment**: - Modified water swallowing test (MWST): 3 mL of cold water to swallow injected onto the floor of the mouth. Score 1 to 5 (1 is inability to swallow with choking and/or breathing changes, and 5 is additional deglutition that occurred more than twice within 30 s. Score ≤ 3: risk of dysphagia. - Cervical auscultation		
Okamoto N. et al. (2015) [26]	**Subjective evaluation**: Do you drop food from your mouth during a meal? Do you feel that food remains in your mouth? Do you choke during a meal? Do you cough during and after a meal?	**Maximum bite force by all dentition** was measured using the Dental Prescale System. The participants bit a pressure-sensitive sheet as hard as possible in the intercuspal position for 3 s. The pressure sensitive sheet showed occlusal contact area and different densities of color depending on the level of the pressure applied. The maximum bite force was determined by the area and density data with a color image scanner. This measurement was taken with and without dentures in participants who wore and did not wear dentures during a meal, respectively.	Results not reported because this variable was not included in the multivariate model.
	**Objective evaluation**: 30 mL water swallowing test (two trained dentists and four dental hygienists, discussion of observations to arrive at a consensus).		
Okamoto N. et al. (2012) [41]	**Subjective evaluation**: Do you drop food from your mouth during a meal? Do you have the feeling that food remains in your mouth? Do you choke during a meal? Do you cough during and after a meal?	- **Maximum bite force** using the Dental Prescale System (FPD-707; Fuji Film Co., Tokyo, Japan). Measurement of maximum bite force in the intercuspal position, a pressure-sensitive sheet (50H; Fuji Film Co.) was inserted into the participant’s mouth and the participant was instructed: “Please bite as hard as possible for 3 s.” The sheet was scanned using the FPD-707 system to analyze the maximum bite force. This measurement was taken with and without dentures in participants who wore and did not wear dentures during a meal, respectively.	Maximum bite force was lower in subjects with swallowing problems compared to those without swallowing problems.
	**Objective evaluation** by two trained dentists and the examiners discussed their observations to achieve a consensus. 30 mL room temperature water swallow test without interruption from a cup in a seated position. Observation of the time needed to drink the water and the presence or absence of choking. Normal: drink water in ≤5 s without interruption or choking. Abnormal: drink water with interruptions or with choking, or take longer than 5 s.		
Nishida T. et al. (2020) [29]	**Subjective evaluation** Have you choked on tea or soup recently? (yes/no)	**Subjective evaluation with one single question on chewing ability**: Can you eat hard foods as well as you could 6 months ago? Answer: impaired/unimpaired	Participants with impaired chewing ability are more prone to present swallowing problems than others.
	**Objective evaluation**: NE		

NE: Not evaluated.

## Data Availability

Not applicable.

## References

[1] Baijens L.W., Clavé P., Cras P., Ekberg O., Forster A., Kolb G.F., Leners J.-C., Masiero S., Mateos-Nozal J., Ortega O. (2016). European Society for Swallowing Disorders—European Union Geriatric Medicine Society White Paper: Oropharyngeal Dysphagia as a Geriatric Syndrome. Clin. Interv. Aging.

[2] Clarfield A.M. (2007). Geriatrics: The Diseases of Old Age and Their Treatment. BMJ.

[3] Serra-Prat M., Hinojosa G., López D., Juan M., Fabré E., Voss D.S., Calvo M., Marta V., Ribó L., Palomera E. (2011). Prevalence of Oropharyngeal Dysphagia and Impaired Safety and Efficacy of Swallow in Independently Living Older Persons. J. Am. Geriatr. Soc..

[4] Cabré M., Serra-Prat M., Force L., Almirall J., Palomera E., Clavé P. (2014). Oropharyngeal Dysphagia Is a Risk Factor for Readmission for Pneumonia in the Very Elderly Persons: Observational Prospective Study. J. Gerontol. A Biol. Sci. Med. Sci..

[5] Carrión S., Cabré M., Monteis R., Roca M., Palomera E., Serra-Prat M., Rofes L., Clavé P. (2015). Oropharyngeal Dysphagia Is a Prevalent Risk Factor for Malnutrition in a Cohort of Older Patients Admitted with an Acute Disease to a General Hospital. Clin. Nutr. Edinb. Scotl..

[6] Lin L.-C., Wu S.-C., Chen H.S., Wang T.-G., Chen M.-Y. (2002). Prevalence of Impaired Swallowing in Institutionalized Older People in Taiwan. J. Am. Geriatr. Soc..

[7] Almirall J., Rofes L., Serra-Prat M., Icart R., Palomera E., Arreola V., Clavé P. (2013). Oropharyngeal Dysphagia Is a Risk Factor for Community-Acquired Pneumonia in the Elderly. Eur. Respir. J..

[8] Ortega O., Martín A., Clavé P. (2017). Diagnosis and Management of Oropharyngeal Dysphagia Among Older Persons, State of the Art. J. Am. Med. Dir. Assoc..

[9] Christmas C., Rogus-Pulia N. (2019). Swallowing Disorders in the Older Population. J. Am. Geriatr. Soc..

[10] Mankekar G. (2015). Swallowing—Physiology, Disorders, Diagnosis and Therapy.

[11] Glick M., Williams D.M., Kleinman D.V., Vujicic M., Watt R.G., Weyant R.J. (2016). A New Definition for Oral Health Developed by the FDI World Dental Federation Opens the Door to a Universal Definition of Oral Health. Br. Dent. J..

[12] Mishellany A., Woda A., Labas R., Peyron M.-A. (2006). The Challenge of Mastication: Preparing a Bolus Suitable for Deglutition. Dysphagia.

[13] Bourdiol P., Hennequin M., Peyron M.-A., Woda A. (2020). Masticatory Adaptation to Occlusal Changes. Front. Physiol..

[14] Peyron M.A., Woda A., Bourdiol P., Hennequin M. (2017). Age-Related Changes in Mastication. J. Oral Rehabil..

[15] Veyrune J.-L., Miller C.C., Czernichow S., Ciangura C.A., Nicolas E., Hennequin M. (2008). Impact of Morbid Obesity on Chewing Ability. Obes. Surg..

[16] Sierpińska T., Gołebiewska M., Długosz J.W. (2006). The Relationship between Masticatory Efficiency and the State of Dentition at Patients with Non Rehabilitated Partial Lost of Teeth. Adv. Med. Sci..

[17] Lamy M., Mojon P., Kalykakis G., Legrand R., Butz-Jorgensen E. (1999). Oral Status and Nutrition in the Institutionalized Elderly. J. Dent..

[18] Cichero J.A.Y. (2018). Age-Related Changes to Eating and Swallowing Impact Frailty: Aspiration, Choking Risk, Modified Food Texture and Autonomy of Choice. Geriatrics.

[19] Sura L., Madhavan A., Carnaby G., Crary M.A. (2012). Dysphagia in the Elderly: Management and Nutritional Considerations. Clin. Interv. Aging.

[20] Levenson S.A., Walker V.L. (2019). It Is Time to Revamp Approaches to Managing Dysphagia in Nursing Homes. J. Am. Med. Dir. Assoc..

[21] Moher D., Shamseer L., Clarke M., Ghersi D., Liberati A., Petticrew M., Shekelle P., Stewart L.A. (2015). PRISMA-P Group Preferred Reporting Items for Systematic Review and Meta-Analysis Protocols (PRISMA-P) 2015 Statement. Syst. Rev..

[22] Cuschieri S. (2019). The STROBE Guidelines. Saudi J. Anaesth..

[23] Page M.J., McKenzie J.E., Bossuyt P.M., Boutron I., Hoffmann T.C., Mulrow C.D., Shamseer L., Tetzlaff J.M., Akl E.A., Brennan S.E. (2021). The PRISMA 2020 Statement: An Updated Guideline for Reporting Systematic Reviews. BMJ.

[24] Tamura F., Mizukami M., Ayano R., Mukai Y. (2002). Analysis of Feeding Function and Jaw Stability in Bedridden Elderly. Dysphagia.

[25] Rech R.S., Baumgarten A., Colvara B.C., Brochier C.W., de Goulart B., Hugo F.N., Hilgert J.B. (2018). Association between Oropharyngeal Dysphagia, Oral Functionality, and Oral Sensorimotor Alteration. Oral Dis..

[26] Okamoto N., Morikawa M., Yanagi M., Amano N., Tomioka K., Hazaki K., Harano A., Kurumatani N. (2015). Association of Tooth Loss With Development of Swallowing Problems in Community-Dwelling Independent Elderly Population: The Fujiwara-Kyo Study. J. Gerontol. A. Biol. Sci. Med. Sci..

[27] Wang T.-F., Chen I.-J., Li I.-C. (2012). Associations between Chewing and Swallowing Problems and Physical and Psychosocial Health Status of Long-Term Care Residents in Taiwan: A Pilot Study. Geriatr. Nurs..

[28] Fukai K., Takiguchi T., Ando Y., Aoyama H., Miyakawa Y., Ito G., Inoue M., Sasaki H. (2011). Critical Tooth Number without Subjective Dysphagia. Geriatr. Gerontol. Int..

[29] Nishida T., Yamabe K., Honda S. (2020). Dysphagia Is Associated with Oral, Physical, Cognitive and Psychological Frailty in Japanese Community-Dwelling Elderly Persons. Gerodontology.

[30] Onodera S., Furuya J., Yamamoto H., Tamada Y., Kondo H. (2016). Effects of Wearing and Removing Dentures on Oropharyngeal Motility during Swallowing. J. Oral Rehabil..

[31] Bomfim F.M.S., Chiari B.M., Roque F.P. (2013). Factors Associated to Suggestive Signs of Oropharyngeal Dysphagia in Institutionalized Elderly Women. CoDAS.

[32] Namasivayam-MacDonald A.M., Morrison J.M., Steele C.M., Keller H. (2017). How Swallow Pressures and Dysphagia Affect Malnutrition and Mealtime Outcomes in Long-Term Care. Dysphagia.

[33] Brochier C.W., Hugo F.N., Rech R.S., Baumgarten A., Hilgert J.B. (2018). Influence of Dental Factors on Oropharyngeal Dysphagia among Recipients of Long-Term Care. Gerodontology.

[34] Furuta M., Komiya-Nonaka M., Akifusa S., Shimazaki Y., Adachi M., Kinoshita T., Kikutani T., Yamashita Y. (2013). Interrelationship of Oral Health Status, Swallowing Function, Nutritional Status, and Cognitive Ability with Activities of Daily Living in Japanese Elderly People Receiving Home Care Services Due to Physical Disabilities. Community Dent. Oral Epidemiol..

[35] Wakabayashi H., Matsushima M., Ichikawa H., Murayama S., Yoshida S., Kaneko M., Mutai R. (2018). Occlusal Support, Dysphagia, Malnutrition, and Activities of Daily Living in Aged Individuals Needing Long-Term Care: A Path Analysis. J. Nutr. Health Aging.

[36] Inui A., Takahashi I., Kurauchi S., Soma Y., Oyama T., Tamura Y., Noguchi T., Murashita K., Nakaji S., Kobayashi W. (2017). Oral Conditions and Dysphagia in Japanese, Community-Dwelling Middle- and Older-Aged Adults, Independent in Daily Living. Clin. Interv. Aging.

[37] Ortega O., Parra C., Zarcero S., Nart J., Sakwinska O., Clavé P. (2014). Oral Health in Older Patients with Oropharyngeal Dysphagia. Age Ageing.

[38] Okabe Y., Takeuchi K., Izumi M., Furuta M., Takeshita T., Shibata Y., Kageyama S., Ganaha S., Yamashita Y. (2017). Posterior Teeth Occlusion and Dysphagia Risk in Older Nursing Home Residents: A Cross-Sectional Observational Study. J. Oral Rehabil..

[39] Ohara Y., Hirano H., Yoshida H., Suzuki T. (2011). Ratio and Associated Factors of Dry Mouth among Community-Dwelling Elderly Japanese Women. Geriatr. Gerontol. Int..

[40] Murakami K., Hirano H., Watanabe Y., Edahiro A., Ohara Y., Yoshida H., Kim H., Takagi D., Hironaka S. (2015). Relationship between Swallowing Function and the Skeletal Muscle Mass of Older Adults Requiring Long-term Care. Geriatr. Gerontol. Int..

[41] Okamoto N., Tomioka K., Saeki K., Iwamoto J., Morikawa M., Harano A., Kurumatani N. (2012). Relationship between Swallowing Problems and Tooth Loss in Community-Dwelling Independent Elderly Adults: The Fujiwara-Kyo Study. J. Am. Geriatr. Soc..

[42] Poisson P., Laffond T., Campos S., Dupuis V., Bourdel-Marchasson I. (2016). Relationships between Oral Health, Dysphagia and Undernutrition in Hospitalised Elderly Patients. Gerodontology.

[43] De Sire A., Ferrillo M., Lippi L., Agostini F., de Sire R., Ferrara P.E., Raguso G., Riso S., Roccuzzo A., Ronconi G. (2022). Sarcopenic Dysphagia, Malnutrition, and Oral Frailty in Elderly: A Comprehensive Review. Nutrients.

[44] Rauen M.S., Moreira E.A.M., Calvo M.C.M., Lobo A.S. (2006). Oral Condition and Its Relationship to Nutritional Status in the Institutionalized Elderly Population. J. Am. Diet. Assoc..

[45] Samnieng P., Ueno M., Shinada K., Zaitsu T., Wright F.A.C., Kawaguchi Y. (2011). Oral Health Status and Chewing Ability Is Related to Mini-Nutritional Assessment Results in an Older Adult Population in Thailand. J. Nutr. Gerontol. Geriatr..

[46] Mesas A.E., Andrade S.M.d., Cabrera M.A.S., Bueno V.L.R.d.C. (2010). Oral Health Status and Nutritional Deficit in Noninstitutionalized Older Adults in Londrina, Brazil. Rev. Bras. Epidemiol. Braz. J. Epidemiol..

[47] Decerle N., Nicolas E., Hennequin M. (2013). Chewing Deficiencies in Adults with Multiple Untreated Carious Lesions. Caries Res..

[48] El Osta N., Hennequin M., Tubert-Jeannin S., Abboud Naaman N.B., El Osta L., Geahchan N. (2014). The Pertinence of Oral Health Indicators in Nutritional Studies in the Elderly. Clin. Nutr..

[49] Godlewski A.E., Veyrune J.L., Nicolas E., Ciangura C.A., Chaussain C.C., Czernichow S., Basdevant A., Hennequin M. (2011). Effect of Dental Status on Changes in Mastication in Patients with Obesity Following Bariatric Surgery. PLoS ONE.

[50] Hennequin M., Mazille M.-N., Cousson P.-Y., Nicolas E. (2015). Increasing the Number of Inter-Arch Contacts Improves Mastication in Adults with Down Syndrome: A Prospective Controlled Trial. Physiol. Behav..

[51] Sabharwal S., Wilson H., Reilly P., Gupte C.M. (2015). Heterogeneity of the Definition of Elderly Age in Current Orthopaedic Research. SpringerPlus.

